# Extraction Efficiency and Alpha-Glucosidase Inhibitory Activities of Green Tea Catechins by Different Infusion Methods

**DOI:** 10.3390/foods12132611

**Published:** 2023-07-06

**Authors:** Tsukasa Orita, Satoshi Chogahara, Mayuko Okuda, Kozue Sakao, Takeshi Miyata, De-Xing Hou

**Affiliations:** 1 Graduate School of Agriculture, Forestry and Fisheries, Kagoshima University, Kagoshima 890-0065, Japan; 2 Tea Division, Kagoshima Prefectural Institute for Agricultural Development, Kagoshima 899-3401, Japan; 3 The United Graduate School of Agricultural Sciences, Kagoshima University, Kagoshima 890-0065, Japan

**Keywords:** green tea, infusion methods, catechins, gallate catechins, alpha-glucosidase

## Abstract

Alpha-glucosidase is an important target for glycemic control with the aim of reducing the risk of type 2 diabetes (T2D). Green tea catechins have been reported to inhibit alpha-glucosidase activity as a potential beverage to control blood glucose levels. However, the effects of the daily infusion style of green tea on tea catechins and their activity remain unclear. In this study, the extraction efficiency of catechins was investigated for 12 green tea extracts (GTEs) infused with 70% ethanol (70% EtOH for 24 h, a favored solvent for catechin extraction), room temperature water infusion (RT H_2_O for 24 h, an easy way to drink tea), and hot water infusion (Hot H_2_O for 90 s, a standard way to drink tea). Eight catechins were quantified by HPLC, and the inhibitory effect of GTEs and their catechins on alpha-glucosidase was measured with both rat intestinal enzymes and human Caco-2 cells. The inhibitory mechanism was further analyzed in silico by docking catechins to human alpha-glucosidase using Molecular Operating Environment software. The results showed that total catechins and gallate catechins were efficiently extracted in the order of 70% EtOH, RT H_2_O, and Hot H_2_O, and the inhibitory activity against alpha-glucosidase also followed a similar order. Pearson correlation analysis indicated that the alpha-glucosidase inhibitory activity of GTEs was significantly positively correlated with the contents of total catechins, especially gallate catechins. Gallate catechins, such as EGCg and ECg, showed lower IC_50_ values than free catechins for the enzyme in both rats and humans. In silico simulation revealed that gallate catechins were bound to the different sites with free catechins, and the docking energy of gallate catechins was lower than that of free catechins. Taken together, our data indicated that the daily infusion style of green tea significantly impacted the extraction efficiency and alpha-glucosidase inhibitory activities of catechins, which will give us insight into the use of green tea catechins for glycemic control through efficient infusion.

## 1. Introduction

Green tea is one of the most popular beverages in the world. In recent years, green tea has been drunk with the expectation of benefits such as antioxidant [1], anti-inflammation [2], anti-obesity [3], and anti-neurodegenerative effects [4]. These beneficial effects of green tea are often dependent on catechins contained in 10–20% of the dry weight of crude tea [5]. Four catechins, such as (−)-epigallocatechin-3-gallate (EGCg), (−)-epigallocatechin (EGC), (−)-epicatechin-3-gallate (ECg), and (−)-epicatechin (EC), are primary constituents of green tea [6]. In particular, EGCg is the most abundant catechin and accounts for 32.8–75.1% of total catechins [7]. On the other hand, (−)-gallocatechin-3-gallate (GCg), (−)-gallocatechin (GC), (−)-catechin-3-gallate (Cg), and (+)-catechin ((+)C) are minor catechins in green tea [6]. According to the presence or absence of gallate groups at the C ring, these eight catechins are divided into gallate catechins (EGCg, ECg, GCg, and Cg) and free catechins (EGC, EC, GC, and (+)C). It has been shown that gallate catechins have stronger bioactivity than free catechins, such as antioxidant [8] and anticancer effects [9]. In particular, EGCg is the most studied catechin and is known to have the strongest bioactivity [10].

Diabetes is a prevalent disease worldwide. It is estimated that 536.6 million people aged 20~79 years are suffering from diabetes, and a total of 6.7 million people died from diabetes in 2021 [11]. Furthermore, the global prevalence of diabetes is estimated to rise to 12.2% (783.2 million) by 2045 [11]. Type 2 diabetes (T2D), formerly called non-insulin-dependent diabetes, accounts for more than 95% of diabetic patients [12] and contributes to the rising prevalence of diabetes. Excessive carbohydrate consumption is one of the causes of T2D. Alpha-glucosidase is an essential enzyme for the final step of carbohydrate digestion and directly contributes to elevating blood glucose levels. In a population with impaired glucose tolerance, administration of the alpha-glucosidase inhibitor acarbose has been reported to reduce the risk of diabetes, suggesting that alpha-glucosidase is an important preventive target for T2D [13]. Alpha-glucosidase is composed of maltase-glucoamylase (MGAM) and sucrase-isomaltase (SI). Each enzyme has a membrane-bound N-terminal subunit (Nt-MGAM and Nt-SI) and a luminal C-terminal subunit (Ct-MGAM and Ct-SI), and each has independent catalytic activity. For instance, Ct-MGAM and Nt-MGAM predominantly hydrolyze oligosaccharides and maltose, respectively [14]. Ct-SI and Nt-SI also have higher activity in sucrose and isomaltose hydrolysis, respectively [14]. However, maltose is hydrolyzed by all four subunits, while sucrose is only hydrolyzed by Ct-SI [15].

In recent years, research on catechins relieving diabetes has become more concerned. The major role of catechins is considered to be inhibiting carbohydrate digestive enzymes, including alpha-amylase and alpha-glucosidase, to decrease fasting blood glucose levels in diabetic models [16]. It has been reported that catechins, especially gallate catechins, strongly inhibit alpha-glucosidase activity in *S. cerevisiae* or a rat model [17,18]. However, the homology of alpha-glucosidase between rats and humans is only 74% [19], and the difference in the inhibitory effect of alpha-glucosidase by the compound was observed between rats and humans [20]. For example, acarbose showed lower inhibitory activity in humans than in rats due to the weak interaction of acarbose with the Nt-MGAM domain [20]. Therefore, the results of studies in rats may not be fully applicable to humans.

On the other hand, the yield and composition of catechins in green tea are greatly affected by the infusion solvent and temperature. Generally, in Japan, crude tea is infused with boiling water at 80–100 °C for a short time or with normal water overnight. It is reported that only 7–8% of the 20% catechin content in dried tea leaves was eluted out when infusing in hot water for 4 min [21]. To obtain more catechins, one of the most reasonable ways is to infuse tea leaves into organic solvents, such as 70% ethanol, which can extract catechins more efficiently.

Kagoshima prefecture is the second largest producer of tea in Japan, and a wide variety of breeds are cultivated due to its warm climate. In particular, the breeds of Yabukita, Saemidori, Yutakamidori, Kuritawase, and Asanoka are encouraged to be cultivated by Kagoshima Prefecture due to their superior growth and tea quality. According to Kagoshima Prefecture’s 2023 data on tea industry promotion measures, the cultivated areas of these tea plantations were Yabukita with 2566 ha (% of total tea plantation area in Kagoshima Prefecture: 31.1%); Saemidori with 1167 ha (14.1%); Yutakamidori with 2202 ha (26.7%); Kuritawase with 25 ha (0.3%); and Asanoka with 286 ha (3.5%). Although the bioactive compounds of typical green tea breeds have been evaluated, the quantitation and bioactivity of bioactive compounds from these local green tea breeds remain unclear. Moreover, the extraction efficiency of the bioactive compounds from daily tea infusion methods is also unclear.

Based on the above information, in this study, we first evaluated the extraction efficiency of total catechins and individual catechins from 12 breeds of local green crude teas using three kinds of infusion methods: (1) daily tea drinking habit infused with 90 °C water for 90 s; (2) easy tea drinking style infused with room temperature (25 °C) water for 24 h; (3) 70% ethanol, a favored solvent for catechin extraction. Then, we compared the inhibitory effects of individual catechins on alpha-glucosidase activity and clarified the relationship between catechins and their inhibitory activity. Moreover, we compared the alpha-glucosidase inhibitory activity of tea catechins between rat and human Caco-2 cells. Finally, the inhibitory mechanism of catechins on human alpha-glucosidase was simulated with in silico molecular docking methods.

## 2. Materials and Methods

### 2.1. Chemicals and Reagents

(−)EC (purity ≥ 99%), (−)EGC (purity ≥ 99%), (+)C (purity ≥ 99%), and (−)ECg (purity ≥ 99%) were purchased from Nagara Science, Co., Ltd. (Gifu, Japan). (−)GCg (purity ≥ 98%) was purchased from Chemodex Ltd. (Gallen, Switzerland). (−)EGCg (purity ≥ 98%) was purchased from Adipogen Life Sciences, Inc. (San Diego, CA, USA). (−)GC (purity ≥ 98%) and (−)Cg (purity ≥ 98%) were purchased from Cayman Chemical Co. (Ann Arbor, MI, USA). Catechol (purity ≥ 99%) and L(+)-ascorbic acid (purity ≥ 99.6%) were purchased from Kanto Kagaku Co., Ltd. (Tokyo, Japan). Maleic acid, gallic acid (GA) (purity ≥ 97%), maltose-monohydrate, Penicillin-Streptomycin-Glutamine (PSG) Mixed Solution, and MEM Non-Essential Amino Acids Solution (100×) were purchased from Nacalai Tesque Inc. (Kyoto, Japan). Disodium maleate, acarbose, and LabAssay^TM^ Glucose were purchased from FUJIFILIM Wako Pure Chemical Co. (Osaka, Japan). Intestinal acetone powder from rats was purchased from Sigma-Aldrich Co., LLC. (Tokyo, Japan). Minimum Essential Medium (MEM) was purchased from Thermo Fisher Scientific, Inc. (Waltham, MA, USA). Fetal bovine serum was purchased from Biowest Co. (Nuaille, France).

### 2.2. Green Tea Extract (GTE) Preparation

Crude tea from the first crop of different breeds was collected from different areas of Kagoshima Prefecture in Japan (Figure S1), and three different infusion methods were performed as follows: (1) daily tea drinking mimic: 1.0 g of crude tea was infused in 50 mL of H_2_O at 90 °C for 90 s by hand stirring for the first 10 s (Hot H_2_O); (2) daily easy drinking mimic: 1.0 g of crude tea was infused in 50 mL of H_2_O at room temperature (25 °C) for 24 h by stirring (RT H_2_O); (3) organic solvent mimic: 1.0 g of crude tea was infused in 50 mL of 70% EtOH at 25 °C for 24 h by stirring (70% EtOH). After infusion, crude tea was separated by centrifugation (2000× *g*, 15 min, 4 °C), and the supernatants (GTEs) were collected by decanting and stored at −20 °C until use.

### 2.3. Catechin Analysis of GTEs by High-Performance Liquid Chromatography (HPLC)

Prior to HPLC analysis, GTEs (infused with Hot H_2_O or RT H_2_O: 1 mL and 70% EtOH: 0.5–1 mL) were transferred to a 5 mL micro-tube, and the volume was filled up to 5 mL with 0.1 mL of 2% catechol solution (containing 2% ascorbic acid) and purified water. A 2% catechol solution was used for the internal standard, and 2% ascorbic acid was used for the stabilization of catechins. Then, the mixtures were filtered through a 0.45 µm PTFE membrane filter (DISMIC-13HP; Toyo Roshi Kaisha, Ltd., Tokyo, Japan) to remove impurities. Catechins of GTEs were analyzed using a Jasco HPLC system (Tokyo, Japan) as described previously [6] with some modification. The HPLC system consisted of an intelligent sampler (AS-2057 Plus), an intelligent column oven (CO-2065 Plus), a quaternary gradient pump (PU-2089 Plus), and a photodiode array detector (MD-2018 Plus). A Cadenza CD-C18 column (3 µm, 150 mm × 4.6 mm; Imtakt Co., Kyoto, Japan) at 40 °C was fitted to the equipment. Mobile phase A was 0.2% (*v*/*v*) phosphoric acid solution, and mobile phase B was methanol and acetonitrile (3:2 *v*/*v*). A gradient elution was performed as follows: 15% phase B (0–7 min), a linear gradient of phase B from 15 to 50% (7–23 min), 100% phase B (23–25 min), and 15% phase B (25–40 min). The flow rate was 1.0 mL/min, and the injection volume was 5 µL. It has been reported that the UV spectrum of each catechin shows strong absorbance between 200 and 220 nm, and a detection wavelength of 210 nm shows an improved signal-to-noise ratio of chromatograms and clear results [6,22]. Therefore, the detection wavelength was set at 210 nm. Each catechin content of GTEs was estimated from the retention time via the standard reference materials and expressed as g/100 g dry leaves.

### 2.4. Measurement of the Inhibitory Activity of GTE and Its Catechins on Rat Alpha-Glucosidase

Inhibitory activity on rat alpha-glucosidase was measured as described previously [23] with some modification. Rat alpha-glucosidase protein solution was extracted from rat intestinal acetone powders using the ultrasonication method. Briefly, 100 mg of intestinal acetone powders from rats were added to 1.0 mL of a 0.1 M maleic acid/disodium maleate buffer solution at pH 6.0. Then, one cycle of 30 s of ultrasonication and 30 s of break were repeated 6 times in ice-cold water, and the residue was separated by centrifugation (3000 rpm, 30 min, 4 °C). The supernatant was collected as the enzyme solution for experiments. Then, 50 µL of 4% maltose (*w*/*v*) and 25 µL of sample solution (GTEs or catechins) were added to a microtube and preincubated at 37 °C for 3 min. After that, 25 µL of the above enzyme solution was added and incubated at 37 °C for 30 min. Consequently, 400 µL of ultrapure water was added and inactivated at 100 °C for 4 min. After 100 µL of the inactivated reaction solution and 150 µL of color reagent (LabAssay™ Glucose kit) were mixed, the glucose contents (mg/dL) were measured at 505 nm through the spectrometer (Infinite 200 Pro MPlex, Tecan Ltd., Männedorf, Switzerland) according to the manufacturer’s instructions with the standard glucose curve. Acarbose, a potent inhibitor of alpha-glucosidase activity, was used as a positive control. The inhibitory activity of each sample was calculated as follows:

Inhibitory activity (%) = 100 − {(*Cs* − *Csb*)/(*Cc* − *Cb*)} × 100

*Cc*: Glucose content of the negative control;
*Cb*: Glucose content of the blank;
*Cs*: Glucose content of the sample;
*Csb*: Glucose content of the sample blank.

### 2.5. Measurement of the Inhibitory Activity of GTE and Its Major Catechins on Alpha-Glucosidase in Human Caco-2 Cells

Inhibitory activity on human alpha-glucosidase was measured using differentiated Caco-2 cells as described previously [19] with some modification. Briefly, Caco-2 cells were purchased from Riken Bioresource Research Center (Tsukuba, Japan) and cultured in Minimal Essential Medium (MEM) supplemented with 20% fetal bovine serum, 1% penicillin-streptomycin-glutamine, and 1% non-essential amino acids at 37 °C in an atmosphere of 5% CO_2_. After passaging, cells (2.0 × 10^4^ cells/well) were seeded in 48-well plates and cultured for 14 days to differentiate into small intestinal epithelial-like cells. After 14 days, cells were washed twice with PBS, and 150 µL of sample solution at each concentration as well as 150 µL of 3 mM maltose were added. Each sample and maltose solution were dissolved in PBS. The cells were further incubated for 2 h at 37 °C in an atmosphere of 5% CO_2_. Then, 100 µL of cell culture supernatant was mixed with 150 µL of color reagent and measured at 505 nm through the spectrometer (Infinite 200 Pro MPlex, Tecan Ltd., Männedorf, Switzerland). The inhibitory activity of the sample was calculated in the same way as in the rat alpha-glucosidase experiment.

### 2.6. Molecular Docking Analysis

Molecular docking analysis was performed to predict the binding site, distance and energy between alpha-glucosidase protein and catechins using ASE-Dock in Molecular Operating Environment (MOE) 2020.06 software (Chemical Computing group Inc., Montreal, QC, Canada). The 3D structures of catechins were obtained from PubChem and the 3D structure (Nt-SI) of human alpha-glucosidase was obtained from Protein Data Bank (PDB ID: 3LPP). Bound water of this protein was removed, hydrogen atoms were added, and their positions were optimized. Consequently, the binding site of the ligand was estimated with a connection distance = 2.0 through Site Finder module of the MOE system and the MMFF94x force field atomic charges were assigned. The ligand was free to move in the binding pocket during docking step. The energies of bound structures were expressed as *Udock* (kcal/mol) which was calculated as follows:

*Udock* = *Uele* + *Uvdw* + *Ustrain*

*Uele*: Coulomb force;
*Uvdw*: Van der Waals force;
*Ustrain*: Difference between the internal energy of the ligand in the complex and the internal energy of the unbound ligand.

### 2.7. Statistical Analysis

The data were shown as mean ± standard deviation (SD). Significant differences between groups of 3 or more were determined by a one-way analysis of variance (ANOVA) test combined with a Tukey–Kramer test. Also, significant differences between the two groups were found by Student’s *t*-test. A probability of *p* < 0.05 was considered significant. A one-way ANOVA test was performed using Graph Pad Prism 9.3.1 (GraphPad software Inc., San Diego, CA, USA). Student’s *t*-test and Pearson correlation analysis were performed using Microsoft Excel software for Microsoft 365 MSO.

## 3. Results

### 3.1. Catechin Extraction Efficiency of GTEs by Different Solvents and Methods

**Figure 1:**
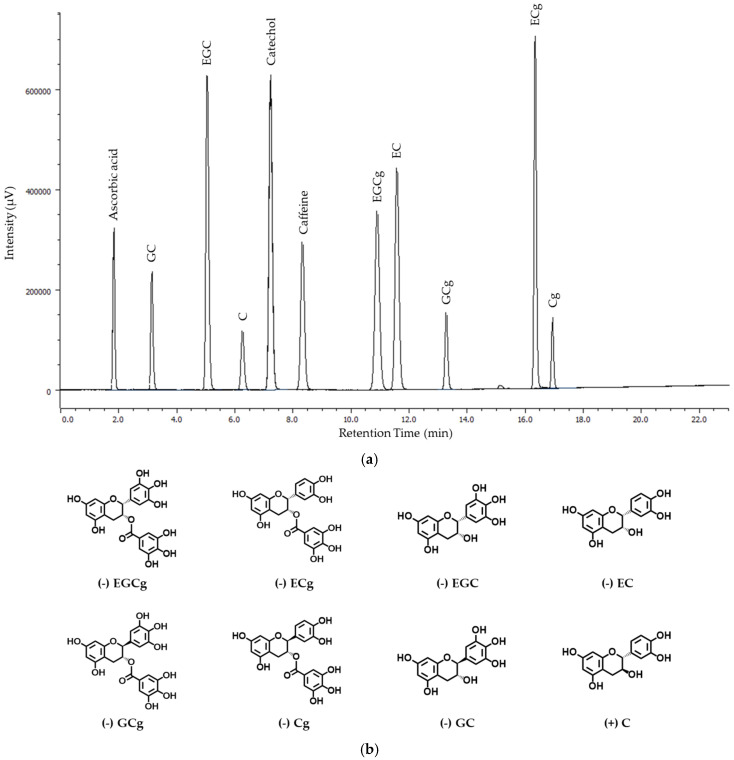
(**a**) HPLC chromatogram profiles of eight catechins as standard reference materials. EGCg: (−)-epigallocatechin-3-gallate; EGC: (−)-epigallocatechin; ECg: (−)-epicatechin-3-gallate; EC: (−)-epicatechin; GCg: (−)-gallocatechin-3-gallate; GC: (−)-gallocatechin; Cg: (−)-catechin-3-gallate; and C: (+)-catechin. (**b**) Chemical structures of eight catechins tested in this study.

Crude tea was infused, respectively, with Hot H_2_O (90 °C, 1.5 min), RT H_2_O (25 °C, 24 h), and 70% EtOH (25 °C, 24 h). Each catechin content of 12 GTEs by three infusion methods was estimated by HPLC. HPLC chromatogram profiles of catechins as reference materials and their chemical structures are shown in Figure 1. The caffeine content of GTEs was not shown in this study.

**Figure 2:**
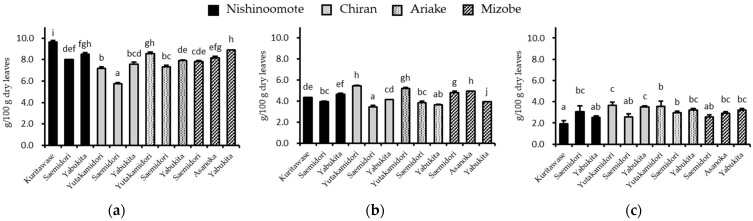
Total catechin content of 12 GTEs infused with (**a**) 70% EtOH (25 °C, 24 h), (**b**) RT H_2_O (25 °C, 24 h), and (**c**) Hot H_2_O (90 °C, 1.5 min). Columns with different letters significantly differ (*p* < 0.05).

Total catechins were efficiently extracted in the order of 70% EtOH, RT H_2_O, and Hot H_2_O with amounts of 7.95 ± 0.112 (Figure 2a and Table S1), 4.36 ± 0.076 (Figure 2b and Table S2), and 2.97 ± 0.25 (Figure 2c and Table S3) g/100 g dry leaves, respectively. In all infusion methods, minor catechins (GCg, GC, and (+)C) accounted for only about 5% of the total catechins, and Cg was undetectable.

A wide range of differences in the total catechin content of GTEs were found in all infusion methods and depended on the production area and breed of green tea. In the infusion method of Hot H_2_O (Table S3), the GTE of the lowest total catechin content was “Kuritawase” from the Nishinoomote area (1.93 ± 0.279 g/100 g dry leaves). The highest content of total catechins was “Yabukita” from the Chiran area (3.64 ± 0.306 g/100 g dry leaves), which was approximately twice as high as the lowest. In the infusion method of RT H_2_O (Table S2), the GTE of “Yabukita” from the Ariake area had the lowest content of total catechins (3.65 ± 0.073 g/100 g dry leaves), and the GTE of “Yutakamidori” from the Chiran area had the highest catechin content (5.46 ± 0.02 g/100 g dry leaves), which was approximately 1.5 times higher than the lowest one. In the infusion method of 70% EtOH (Table S1), the lowest content of catechin was “Saemidori” from the Chiran area (5.75 ± 0.084 g/100 g dry leaves), and the highest was “Kuritawase” from the Nishinoomote area (9.62 ± 0.181 g/100 g dry leaves), which was approximately 1.7 times higher than the lowest one. It was noted that “Saemidori” showed a lower content of total catechins in all infusion methods.

**Figure 3:**
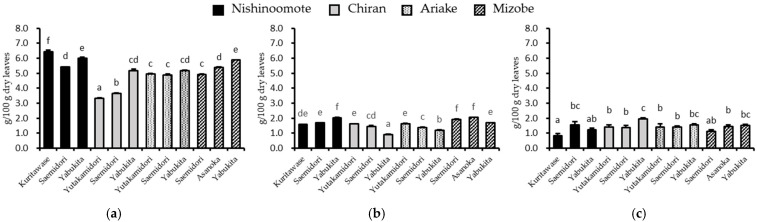

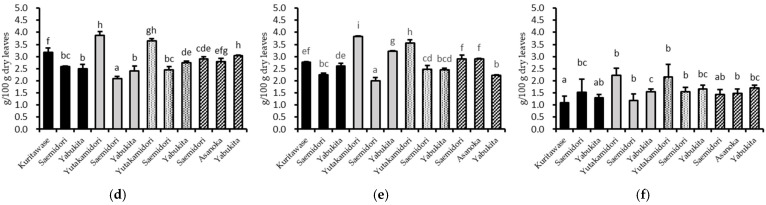
Gallate catechins content of 12 GTEs infused with (**a**) 70% EtOH (25 °C, 24 h), (**b**) RT H_2_O (25 °C, 24 h), and (**c**) Hot H_2_O (90 °C, 1.5 min). Free catechins content of 12 GTEs infused with (**d**) 70% EtOH (25 °C, 24 h), (**e**) H_2_O (25 °C, 24 h), and (**f**) H_2_O (90 °C, 1.5 min). Columns with different letters significantly differ (*p* < 0.05).

**Figure 4:**
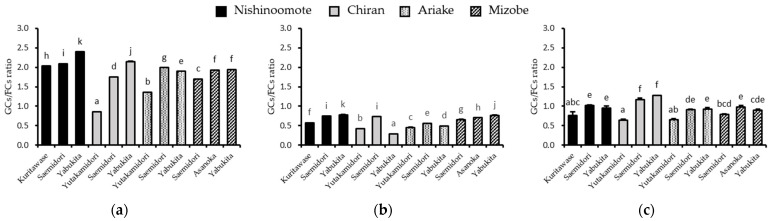
Ratio of gallate catechins/free catechins (GCs/FCs ratio) of 12 GTEs infused with (**a**) 70% EtOH (25 °C, 24 h), (**b**) RT H_2_O (25 °C, 24 h), and (**c**) Hot H_2_O (90 °C, 1.5 min). Columns with different letters significantly differ (*p* < 0.05).

The contents of gallate catechins (GCs: total amount of ECg, EGCg, and GCg) and free catechins (FCs: total amount of EC, EGC, (+)C, and GC) in the GTEs varied widely among different infusion solvents or methods (Figure 3 and Figure 4 and Tables S1–S3). In GTEs infused with Hot H_2_O, the mean amounts of GCs and FCs were 1.36 ± 0.293 and 1.57 ± 0.316 g/100 g dry leaves, respectively, and the mean ratio of GCs/FCs was 1.09 (Figure 4c). In GTEs infused with RT H_2_O, the mean amounts of GCs and FCs were 1.57 ± 0.316 and 2.83 ± 0.557 g/100 g dry leaves, respectively, and the mean ratio of GCs/FCs was 0.72 (Figure 4b). Notedly, in GTEs infused with 70% EtOH, the mean amounts of GCs and FCs were 5.06 ± 0.830 and 2.95 ± 0.594 g/100 g dry leaves, respectively, and the mean ratio of GCs/FCs was 2.21 (Figure 4a). The data revealed that infusing with 70% EtOH could obtain the highest gallate catechins amount and the highest ratio of GCs/FCs.

### 3.2. Inhibitory Activity of GTEs and Its Catechins on Rat Alpha-Glucosidase

The inhibitory activity of GTEs on rat alpha-glucosidase was measured using maltose as a substrate. GTEs from all infusion methods were diluted two-fold to measure the inhibitory activity of GTEs infused using the three methods. To confirm whether the remaining EtOH from four-fold dilution influences the enzyme activity, we measured the amount of glucose produced by alpha-glucosidase in both water solution (negative control) and 4-fold dilution solution from 70% EtOH. No change in the amount of glucose was observed between these two solutions.

As shown in Table 1, GTEs infused with 70% EtOH showed the most inhibitory effect on rat alpha-glucosidase activity, followed by GTEs infused with RT H_2_O and Hot H_2_O. Moreover, significant differences were also observed in the production area and breeds of green tea. These data indicated that the inhibitory activity may be correlated with the total catechin contents of GTEs (Tables S1–S3). Thus, we used Pearson’s correlation analysis to calculate the correlation coefficient between individual catechin content and rat alpha-glucosidase inhibitory activity. As shown in Table 2, gallate catechins (total of ECg, EGCg, and GCg) in three infusion methods revealed a significantly high positive correlation, while free catechins (total of EC, EGC, (+)C, and GC) had no significant correlation with inhibitory activity on rat alpha-glucosidase. Furthermore, a significant correlation of rat alpha-glucosidase inhibitory activity was observed in total catechins infused with Hot H_2_O and 70% EtOH, which contained a higher ratio of gallate catechins than the GTEs infused with RT H_2_O. This result suggests that gallate catechins were the principal components of the inhibitory activity of rat alpha-glucosidase of GTEs.

**Figure 5:**
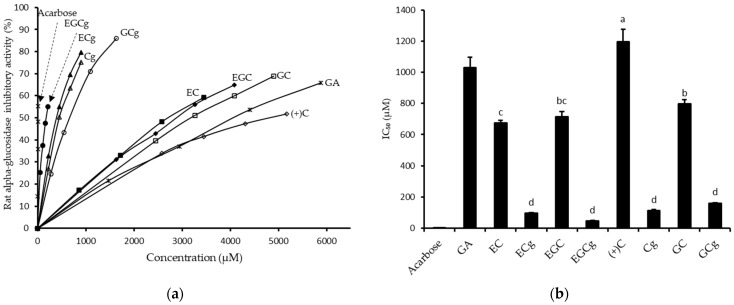
Rat alpha-glucosidase inhibitory activity of catechins. Acarbose was a positive control. (**a**) Inhibitory percentages by different concentrations of each catechin. (**b**) Inhibitory concentrations of the half-activity (IC_50_) of each catechin. Columns with different letters in the catechins group significantly differ (*p* < 0.05).

To further determine the inhibitory effect of individual catechins, the inhibitory activities of eight catechins were investigated at four defined concentrations. Acarbose (a strong inhibitor of alpha-glucosidase) and GA (a weak inhibitor of alpha-glucosidase) were used as controls. The concentration course of catechins on the inhibitory activity of rat alpha-glucosidase is shown in Figure 5a. The half maximal inhibitory concentration (IC_50_) was calculated from the concentration course and shown in Figure 5b. The order of inhibitory activity (IC_50_ values) was EGCg (46.0 ± 3.73 µM), ECg (96.9 ± 4.5 µM), Cg (112.5 ± 7.8 µM), GCg (160.9 ± 2.4 µM), EC (675.0 ± 15.7 µM), EGC (713.3 ± 35.3 µM), GC (798.3 ± 26.8 µM), and (+)C (1196.6 ± 79.6 µM). As a control, IC_50_ values for acarbose and GA were 2.17 ± 0.23 µM, and 1031.50 ± 64.48 µM, respectively. These data indicated that gallate catechins, including ECg, EGCg, Cg, and GCg, were the principal components of the inhibitory activity rather than non-gallate-type catechins.

### 3.3. Inhibitory Activity of GTE and Its Catechins on Human Alpha-Glucosidase

Although the inhibitory effects of tea catechins on alpha-glucosidase were validated in rat alpha-glucosidase as undertaken in most studies [17,18], the homology of alpha-glucosidase between rats and humans is only 74% [19], which affects the inhibitory effect of the compounds [20]. For this reason, we further investigated the inhibitory activity of GTEs and their catechins on human alpha-glucosidase in differentiated Caco-2 cells.

**Figure 6:**
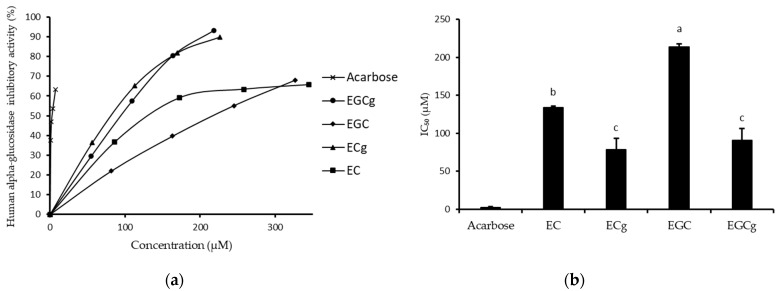
The inhibitory activity of major catechins on human alpha-glucosidase. Acarbose was used as a positive control. (**a**) Inhibitory percentages by defined concentrations of catechins. (**b**) Inhibitory concentrations of the half-activity (IC_50_) of major catechins. Columns with different letters in the major catechins group significantly differ (*p* < 0.05).

The inhibitory activities of the major four catechins on human alpha-glucosidase were verified in differentiated Caco-2 cells with a maltose substrate. As shown in Figure 6a, human alpha-glucosidase activity was inhibited by four catechins in a concentration-dependent manner. Gallate catechins such as ECg and EGCg (IC_50_ = 78.4 ± 15.2 and 90.7 ± 15.8 µM) strongly inhibited human enzyme activity compared to their free forms such as EC and EGC (IC_50_ = 133.7 ± 2.2 and 213.5 ± 3.8 µM) (Figure 6b). As a control, acarbose showed the strongest inhibitory effect (IC_50_ = 2.56 ± 0.62 µM). Interestingly, ECg (IC_50_ = 78.4 ± 15.2 µM) showed stronger inhibitory activity than EGCg (IC_50_ = 90.7 ± 15.8 µM) on human alpha-glucosidase, which is in contrast to the results from rat alpha-glucosidase inhibitory activity by EGCg and ECg.

**Figure 7:**
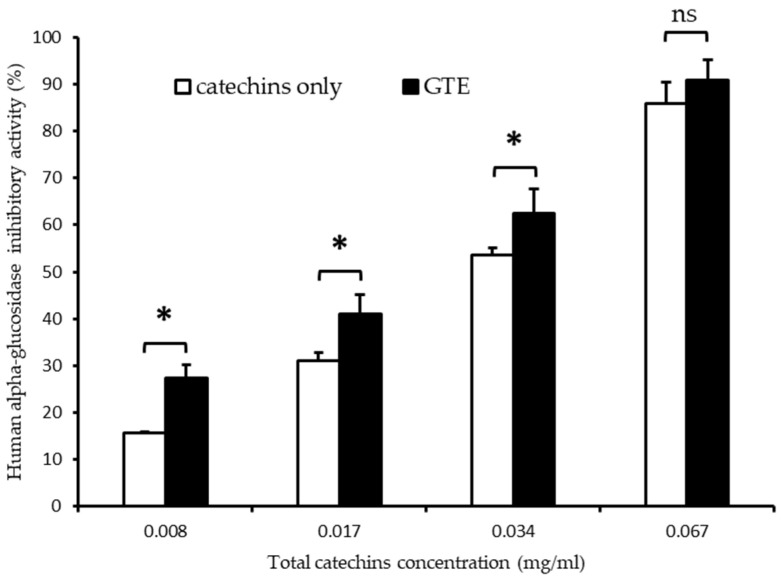
Inhibitory activities of reconstituted catechins and GTE on human alpha-glucosidase. “Yabukita” from the Nishinoomote area infused with Hot H_2_O (90 °C, 1.5 min) was used as the GTE in this experiment. * *p* < 0.05 was significantly different in the same concentration of catechins. ns was not significantly different (*p* < 0.05).

In order to confirm the weight of catechins in whole GTE on the inhibition of human alpha-glucosidase, we mimicked tea catechin content by reconstituting seven catechins with concentrations equal to those in the GTE and then compared the inhibitory activity with whole GTE (Figure 7). “Yabukita” tea from the Nishinoomote area infused with Hot H_2_O was chosen as a typical GTE. As shown in Figure 7, both reconstituted catechins and GTE inhibited human alpha-glucosidase in a similar concentration-dependent manner. The IC_50_ values for reconstituted catechins (IC_50_ = 0.030 ± 0.002 mg/mL) and for GTE (IC_50_ = 0.023 ± 0.004 mg/mL) were different. The reconstituted catechins were estimated to have a 76% weight of GTE on the inhibitory activity of human alpha-glucosidase.

### 3.4. Prediction of Binding Site, Energy, and Distance of Catechins Binding to Human Alpha-Glucosidase and Catechins by MOE-ASEDock

**Figure 8:**
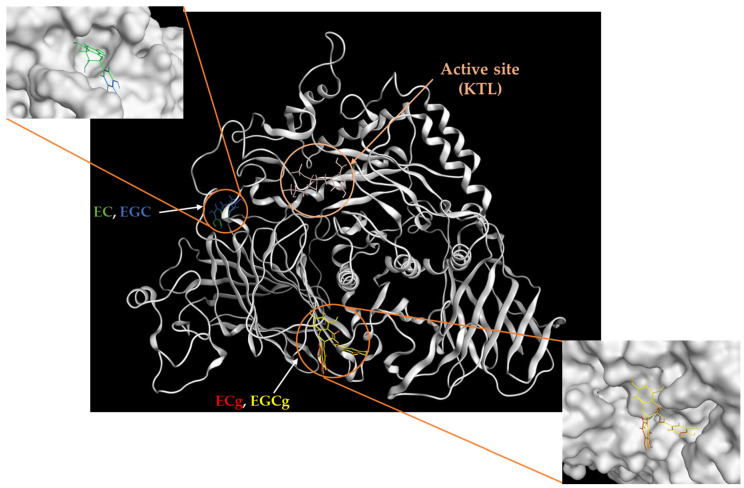
In silico binding site of EC (green), EGC (blue), ECg (red), and EGCg (yellow) in the Nt-SI protein (PDB ID: 3LPP). Kotalanol (KTL) was used as a control.

**Figure 9:**
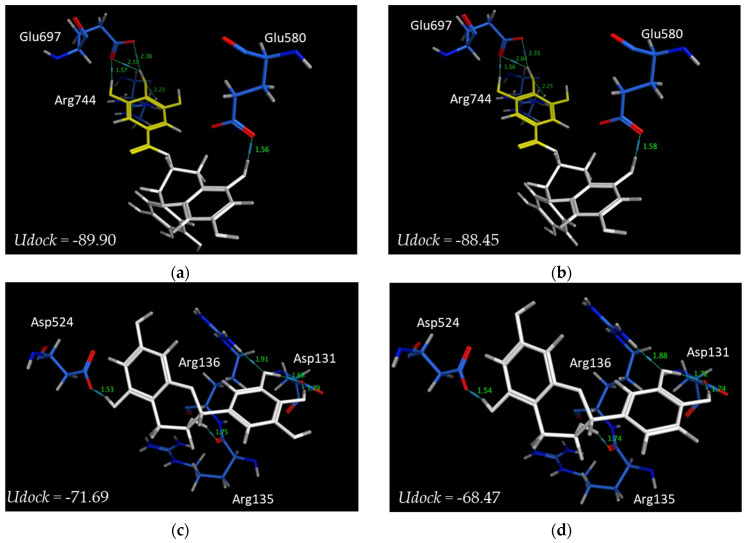
Interaction of catechins with amino acid residues of proteins at binding sites. (**a**) EGCg and (**b**) ECg are represented by white and yellow (gallate group) skeletons, and (**c**) EGC and (**d**) EC are represented by white skeletons. The green and blue dot lines in each figure indicate H-bond interactions, and the green letters indicate bound distance.

In order to further elucidate the properties of catechins inhibiting human alpha-glucosidase activity, we performed molecular docking simulations based on the structures of human alpha-glucosidase and catechins using MOE-ASEDock software. The binding site, energy, and distance of the catechins to the Nt-SI protein were predicted. Kotalanol, a potent alpha-glucosidase inhibitor, is reported to bind with the active site of Nt-SI, forming hydrogen bonds with the active residues Asp472 and Asp571 by X-ray diffraction. Our data revealed that all catechins (EC, EGC, ECg, and EGCg) did not bind to the active site. Furthermore, the binding sites differed between gallate catechins (EGCg and ECg) and free catechins (EGC and EC) (Figure 8). Gallate catechins bound to the protein pocket have a lower energy than free catechins. The binding energy (*Udock*) of EGCg, ECg, EGC, or EC was −89.9, −88.5, −71.7, or −68.5 kcal/mol, respectively (Figure 9). In the interaction of catechins with amino acid residues of binding sites, the hydroxyl group of gallate catechins formed five hydrogen bonds with Arg744, Glu697, and Glu580. In particular, the hydroxyl group of the gallate group had four hydrogen bonds. On the other hand, the hydroxyl group of free catechins formed five hydrogen bonds with other amino acid residues (Asp131, Arg135, Arg136, and Asp524). These docking results may support why gallate catechins have stronger inhibitory activity than free catechins on human alpha-glucosidase.

## 4. Discussion

In this study, the total contents and individual compositions of catechins from 12 breeds of crude tea were investigated by three different infusion methods. We found that the amounts and compositions of catechins were significantly different among infusion methods, although the difference was also observed between green tea breeds. These differences impacted the inhibitory effect of green tea extracts on alpha-glucosidase activity. The significant difference in total catechin contents was first observed in tea breeds rather than production areas. “Saemidori” had a lower catechin content than other breeds in the same production area (Figure 2). Although Saemidori’s low catechin content has been reported as a breed characteristic [24], flavonols such as quercetin glycosides are rich in this breed [25]. These provide insight into using green tea breeds for health benefits.

The most significant differences were observed between different infusion methods. In particular, the infusion solvent, temperature, and time had significant impacts on the yield and composition of catechins from crude tea. The extraction efficiency is summarized in Table 3. Infusing with Hot H_2_O for 90 s, a daily drinking habit, could extract 2.97 ± 0.506 g/100 g dry leaves of total catechins and 1.36 ± 0.293 g/100 g dry leaves of gallate catechins. Infusing with RT H_2_O for 24 h, an easy way of drinking tea, resulted in 4.36 ± 0.642 g/100 g dry leaves of total catechins and 1.57 ± 0.316 g/100 g dry leaves of gallate catechins. On the other hand, infusing with 70% EtOH, a favored solvent for catechin extraction, obtained 7.95 ± 0.970 g/100 g dry leaves of total catechins and 5.06 ± 0.83 g/100 g dry leaves of gallate catechins, which were 2.7 and 3.7 times higher than those infused with Hot H_2_O, a daily drinking habit (Table 3). Accordingly, the order of the inhibitory effect on alpha-glucosidase activity was 70% EtOH, RT H_2_O, and Hot H_2_O (Table 3). It was reported that each cup of tea (200 cc) contained approximately 117.2 mg of catechins in this study, and diabetic patients who drank green tea containing 582.8 mg of catechins per day could lower serum glucose levels by 8.9 mg/dL [26]. From this point, our data revealed that drinking five cups of green tea per day from Hot H_2_O or three cups of green tea per day from RT H_2_O can meet the requirement. It is noticed that infusing with 70% EtOH obtained the highest amount of total catechins and gallate catechins, although it is not usually used in daily drinking. However, it will give us insight into how to prevent fatty livers in people who usually drink alcohol. A project to infuse green tea into distilled spirits is currently underway.

Alpha-glucosidase is one of the major potential preventive targets of T2D [27], and many studies have been reported on its inhibitory activity by plant bioactive components [17]. In this study, GTEs and their bioactive components, catechins, inhibit alpha-glucosidase activity in both rats and humans. It has been reported that gallate catechins strongly inhibit alpha-glucosidase activity in S. cerevisiae or rats [17,18], and this study is consistent with those reports. Gallate catechins showed a stronger inhibitory effect on human alpha-glucosidase. Interestingly, EGCg showed the strongest inhibitory activity on rat alpha-glucosidase, while the strongest inhibitor on alpha-glucosidase from human Caco-2 cells was ECg, not EGCg. One of the possible reasons for these results is the difference in alpha-glucosidase subunit composition between rats and humans. MGAM and SI are present in the human intestinal tract in a ratio of 1:19 [28], and MGAM is virtually absent in the Caco-2 cells used in this study [29]. It has been reported that catechins selectively inhibit the individual subunits of alpha-glucosidase [30]. For example, ECg had the highest inhibitory activity on Ct-SI and Nt-SI of the four catechins tested [31], which supports the results in this study. Moreover, ECg is reported to have the strongest inhibitory effect on the absorption of glucose in the intestine [32] and to be more stable than EGCg [33]. These data suggest that ECg contained in green tea is a potential stronger compound for the initial prevention of T2D against excessive consumption of starch. However, MGAM is actually present in small amounts in the human tract [34] and contributes 20% of maltase activity. Especially, Ct-MGAM showed the strongest maltase activity than other subunits [35]. Therefore, the human model, which has MGAM subunits, is needed to clarify the true effects of catechins on human alpha-glucosidase.

It is interesting that the catechin mixture reconstituted in equal concentrations of catechins to the GTE only showed approximately 76% of the inhibitory activity of GTE by IC_50_ values (Figure 7). It is known that kaempferol, quercetin, and myricetin glycosides are involved in green tea at 0.5–2.5% of dry crude tea weight [36] and have potent alpha-glucosidase inhibitory activity [37,38,39]. Therefore, these flavonoids in GTE also contribute to the alpha-glucosidase inhibitory effect, although catechins are major components in green tea.

The stability of catechins in the gastrointestinal tract is an important factor in discussing whether catechins are truly effective on alpha-glucosidase in vivo. Although catechins are stable under acidic conditions, weak alkaline conditions cause autoxidation of catechins [33]. In the human organism, the pH range has been reported to be acidic in the stomach, 6.4–6.6 in the duodenum and proximal small intestine, and 7.3–7.5 in the distal small intestine [40,41]. Alpha-glucosidase activity is reported to be eight times higher in the proximal jejunum than in the ileum [42], and glucose absorption ability is highest in the proximal jejunum [43]. Therefore, catechins are expected to be better potential compounds to have an anti-diabetic effect in the intestinal tract.

For in silico simulation, Nt-SI was used for molecular docking with catechins because MGAM is virtually absent in Caco-2 cells [29] and Ct-SI was not yet structurally determined. Gallate catechins were observed to bind the sites differently from those of free catechins and had a lower binding energy expressed as *Udock* than free catechins. These results may contribute to the stronger inhibitory effect of gallate catechins in Caco-2 cell experiments. To clarify in more detail why gallate catechins have strong inhibitory activity, molecular dynamics simulations for predicting structural changes in the active site by binding catechins are needed [30,44]. On the other hand, (+)C and EGCg are also reported to bind to the active site of human Nt-MGAM or Ct-MGAM [15,45]. Therefore, the different binding sites of catechins to each subunit may be related to the selective inhibition of catechins against alpha-glucosidase activity.

## 5. Conclusions

The extraction efficiency of green tea catechins as well as their inhibitory activities on alpha-glucosidase were high, in the order of 70% EtOH, RT H_2_O, and Hot H_2_O. The extracted gallate catechins had stronger enzyme inhibition than free catechins. It is possible that gallate catechins can easily and stably bind to alpha-glucosidase due to their low docking energy. Therefore, the daily infusion style of green tea significantly impacts the extraction efficiency and alpha-glucosidase inhibitory activities of catechins, which will give insight into how to use green tea catechins for glycemic control through efficient infusion.

## Figures and Tables

**Table 1:** The inhibitory activity on rat alpha-glucosidase by 12 GTEs infused with 70% EtOH (25 °C, 24 h), RT H_2_O (25 °C, 24 h), and Hot H_2_O (90 °C, 1.5 min) ^(1)^.

Area	Breed	Inhibitory Activity (%)		
		70% EtOH (25 °C, 24 h)	H_2_O (25 °C, 24 h)	H_2_O (90 °C, 1.5 min)
Nishinoomote	Kuritawase	97.0 ± 0.6 c	91.1 ± 2.9 c	72.9 ± 0.9 a
	Saemidori	92.6 ± 1.9 b	92.2 ± 1.6 c	81.6 ± 0.4 cde
	Yabukita	96.5 ± 0.8 c	92.9 ± 1.4 c	78.1 ± 0.4 c
Chiran	Yutakamidori	82.5 ± 1.9 a	91.3 ± 0.7 c	77.9 ± 0.7 c
	Saemidori	86.3 ± 2.0 a	81.4 ± 3.2 ab	79.0 ± 0.5 cd
	Yabukita	93.1 ± 1.0 bc	79.8 ± 7.0 a	84.9 ± 0.3 e
Ariake	Yutakamidori	94.4 ± 0.7 bc	89.5 ± 1.1 bc	80.0 ± 0.3 cd
	Saemidori	95.1 ± 0.6 bc	86.7 ± 0.9 abc	83.2 ± 0.9 de
	Yabukita	95.4 ± 0.2 bc	91.1 ± 1.0 c	79.3 ± 1.7 cd
Mizobe	Semidry	93.8 ± 0.3 bc	92.9 ± 1.0 c	73.1 ± 2.4 ab
	Asanoka	95.4 ± 0.3 bc	92.3 ± 1.2 c	78.5 ± 2.2 cd
	Yabukita	96.3 ± 0.1 bc	88.4 ± 1.4 abc	77.8 ± 2.1 bc
Mean ± SD		93.2 ± 4.21 a	89.1 ± 4.23 a	78.7 ± 3.37 b

^(1)^ GTEs of all infusion methods were diluted two-fold to measure the inhibitory activity of GTEs infused using the three methods. The numerical values with different letters significantly differ (*p* < 0.05) in the same column. In the “Mean ± SD” row, the numerical values with different letters significantly differ (*p* < 0.05).

**Table 2:** Pearson’s correlation coefficients between each catechin content (g/100 g dry leaves) and rat alpha-glucosidase inhibitory activity (%).

Pearson’s Correlation Coefficient			
	Each Infusion Method		
Catechins	70% EtOH (25 °C, 24 h)	H_2_O (25 °C, 24 h)	H_2_O (90 °C, 1.5 min)
EC	−0.45	0.31	0.13
EGC	−0.24	−0.01	0.21
(+)C	−0.53	0.44	0.30
GC	−0.48	0.13	0.36
Free form	−0.29	0.15	0.35
ECg	0.74 **	0.78 *	0.92 ***
EGCg	0.83 ***	0.69 *	0.95 ***
GCg	−0.11	−0.25	0.63 *
Gallate form	0.83 ***	0.71 **	0.95 ***
Total catechins	0.61 *	0.49	0.74 *

* *p* < 0.05, ** *p* < 0.01, and *** *p* < 0.001 indicate significant Pearson correlation.

**Table 3:** Summary of catechins extraction efficiency and alpha-glucosidase inhibitory activity of GTEs by three infusion methods.

	H_2_O (90 °C, 1.5 min)	H_2_O (25 °C, 24 h)	70% EtOH (25 °C, 24 h)
Total catechins (g/100 g dry leaves)	2.97 ± 0.506 a	4.36 ± 0.642 b	7.95 ± 0.970 c
Gallate catechins (g/100 g dry leaves)	1.36 ± 0.293 a	1.57 ± 0.316 a	5.06 ± 0.830 b
Free catechins (g/100 g dry leaves)	1.57 ± 0.313 a	2.83 ± 0.553 a	2.95 ± 0.594 b
GCs/FCs ratio	0.90 a	0.58 b	1.79 c
Alpha-glucosidase inhibitory activity (%)	78.7 ± 3.37 a	89.1 ± 4.23 b	93.2 ± 4.21 b

The numerical values with different letters significantly differ (*p* < 0.05) in the same row.

## Data Availability

Data is contained within the article.

## Supplementary Materials

The following supporting information can be downloaded at: https://www.mdpi.com/article/10.3390/foods12132611/s1.
