# Supplementary material for: Extraction Efficiency and Alpha-Glucosidase Inhibitory Activities of Green Tea Catechins by Different Infusion Methods
Source: Foods. 2023 Jul 6;12(13):2611. doi: 10.3390/foods12132611 (PMC10340592; doi:10.3390/foods12132611)
Supplement: Supplementary file 1 [file foods-12-02611-s001.zip › foods-2465589-supplementary.pdf]

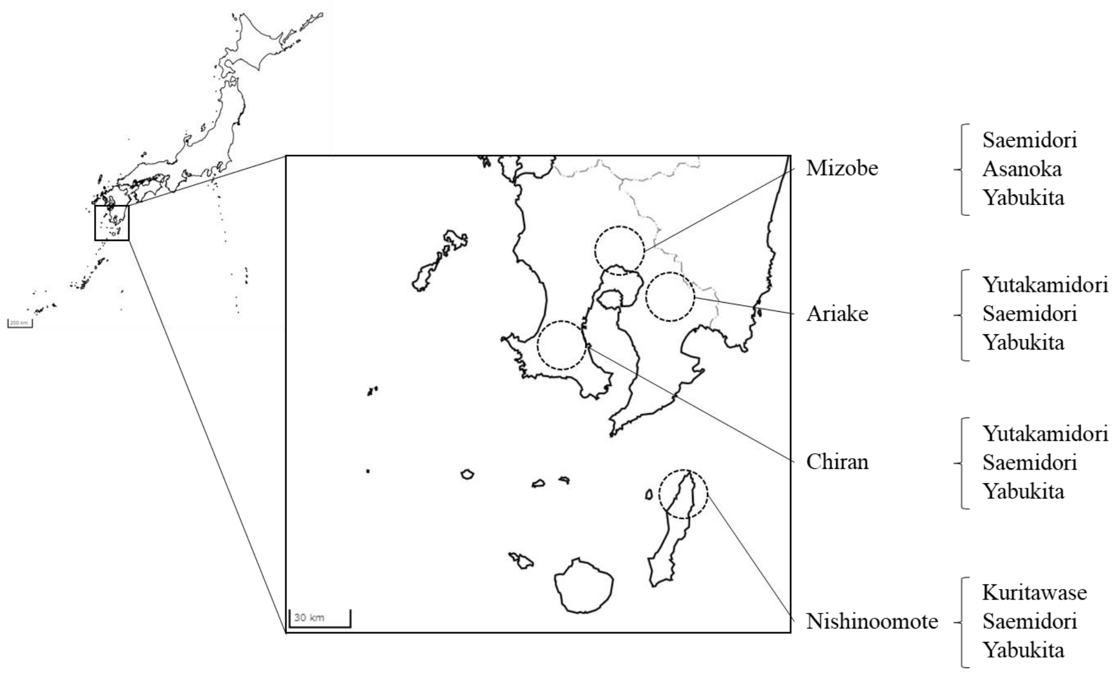

Figure S1. Geographic origins of Kagoshima prefecture and breeds of green tea leaves used in this study. The map of Kagoshima was taken from the Geospatial Information Authority of Japan.

Table S1. Catechin content of 12 green tea breeds infused by 70% EtOH (25 °C, 24 h). The numerical values with different letters significantly differ ($p < 0.05$) in the same column. In "Means ± SD" row, the numerical values with different letters significantly differ ($p < 0.05$).

**Area**	**Breed**	**Gallate catechins**				**Free catechins**					**Total catechins**	**GC/FC**
		ECg	EGCg	GCg	Total	EC	EGC	(+)C	GC	Total		
Nishinoomote	Kuritawase	1.20 ± 0.018i	5.21 ± 0.098h	0.03 ± 0.002ab	6.44 ± 0.081f	0.82 ± 0.015e	2.12 ± 0.035f	0.12 ± 0.013b	0.12 ± 0.004bc	3.17 ± 0.048f	9.62 ± 0.181i	2.03 ± 0.005h
	Saemidori	0.91 ± 0.005ef	4.39 ± 0.006f	0.12 ± 0.004g	5.42 ± 0.010d	0.57 ± 0.004ab	1.81 ± 0.004cd	0.09 ± 0.005ab	0.12 ± 0.004cd	2.59 ± 0.000bc	8.01 ± 0.015def	2.09 ± 0.003i
	Yabukita	1.04 ± 0.024h	4.91 ± 0.095g	0.04 ± 0.001bc	5.99 ± 0.083e	0.59 ± 0.009ab	1.73 ± 0.031c	0.10 ± 0.007ab	0.08 ± 0.005a	2.50 ± 0.036b	8.49 ± 0.169fgh	2.4 ± 0.0001k
Chiran	Yutakamidori	0.57 ± 0.013a	2.67 ± 0.064a	0.08 ± 0.001ef	3.32 ± 0.055a	0.99 ± 0.023f	2.51 ± 0.050g	0.16 ± 0.007c	0.20 ± 0.008g	3.86 ± 0.063h	7.18 ± 0.167b	0.86 ± 0.000a
	Saemidori	0.67 ± 0.009b	2.93 ± 0.041b	0.06 ± 0.002d	3.66 ± 0.036b	0.57 ± 0.007ab	1.32 ± 0.017a	0.11 ± 0.005ab	0.09 ± 0.003a	2.09 ± 0.023a	5.75 ± 0.084a	1.75 ± 0.002d
	Yabukita	0.86 ± 0.017de	4.2 ± 0.102def	0.12 ± 0.005g	5.18 ± 0.088cd	0.59 ± 0.014ab	1.57 ± 0.036b	0.11 ± 0.005ab	0.14 ± 0.008de	2.41 ± 0.044b	7.60 ± 0.187bcd	2.15 ± 0.0030j
Ariake	Yutakamidori	0.80 ± 0.010c	4.05 ± 0.054cde	0.09 ± 0.001f	4.93 ± 0.047c	0.79 ± 0.013e	2.57 ± 0.016g	0.12 ± 0.007ab	0.16 ± 0.006f	3.64 ± 0.030g	8.57 ± 0.108gh	1.35 ± 0.002b
	Saemidori	0.85 ± 0.013cd	3.97 ± 0.066cd	0.07 ± 0.002de	4.89 ± 0.057c	0.54 ± 0.007a	1.72 ± 0.027c	0.09 ± 0.003a	0.10 ± 0.002ab	2.46 ± 0.027b	7.34 ± 0.119bc	1.99 ± 0.001g
	Yabukita	0.84 ± 0.015cd	4.26 ± 0.043ef	0.08 ± 0.004ef	5.18 ± 0.038cd	0.59 ± 0.006b	1.91 ± 0.022de	0.10 ± 0.005ab	0.13 ± 0.006cde	2.73 ± 0.012cd	7.91 ± 0.07de	1.89 ± 0.005e
Mizobe	Saemidori	0.96 ± 0.013fg	3.90 ± 0.059c	0.05 ± 0.003c	4.91 ± 0.049c	0.73 ± 0.014d	1.96 ± 0.028e	0.10 ± 0.007ab	0.10 ± 0.003ab	2.90 ± 0.022de	7.81 ± 0.101cde	1.7 ± 0.004c
	Asanoka	1.01 ± 0.015gh	4.32 ± 0.060f	0.04 ± 0.002c	5.37 ± 0.055d	0.70 ± 0.010cd	1.75 ± 0.023c	0.19 ± 0.011c	0.14 ± 0.007ef	2.79 ± 0.035d	8.16 ± 0.128efg	1.93 ± 0.005f
	Yabukita	0.97 ± 0.002g	4.87 ± 0.019g	0.03 ± 0.001a	5.87 ± 0.014e	0.69 ± 0.002c	2.15 ± 0.009f	0.10 ± 0.005ab	0.10 ± 0.005a	3.03 ± 0.001ef	8.90 ± 0.021h	1.94 ± 0.004f
Means ± SD		0.89±0.169	4.14 ± 0.745	0.07 ± 0.032	5.06 ± 0.83	0.68 ± 0.134	1.93 ± 0.365	0.12 ± 0.029	0.12 ± 0.035	2.95 ± 0.594	7.95 ± 0.970	1.84 ± 0.384

Table S2. Catechin content of 12 green tea breeds infused by RT H_2O (25 °C, 24 h). The numerical values with different letters significantly differ ($p < 0.05$) in the same column. In "Means ± SD" row, the numerical values with different letters significantly differ ($p < 0.05$).

Area	Breed	Gallate catechins				Free catechins					Total catechins	GC/FC
		ECg	EGCg	GCg	Total	EC	EGC	(+)C	GC	Total		
Nishinoomote	Kuritawase	0.26 ± 0.001ef	1.33 ± 0.006de	0.00 ± 0.005ab	1.59 ± 0.000de	0.68 ± 0.004e	1.90 ± 0.007ef	0.08 ± 0.002bcde	0.12 ± 0.001e	2.77 ± 0.011ef	4.36 ± 0.015de	0.57 ± 0.002f
	Saemidori	0.23 ± 0.005d	1.43 ± 0.019ef	0.03 ± 0.000gh	1.68 ± 0.017e	0.45 ± 0.006a	1.64 ± 0.023bc	0.06 ± 0.000a	0.11 ± 0.003de	2.26 ± 0.023bc	3.94 ± 0.056bc	0.74 ± 0.000i
	Yabukita	0.27 ± 0.010f	1.75 ± 0.044i	0.01 ± 0.007abc	2.03 ± 0.033f	0.62 ± 0.014b	1.82 ± 0.046de	0.09 ± 0.005de	0.08 ± 0.005ab	2.60 ± 0.050de	4.63 ± 0.117ef	0.78 ± 0.002k
Chiran	Yutakamidori	0.23 ± 0.003d	1.36 ± 0.003def	0.04 ± 0.000h	1.63 ± 0.000e	0.83 ± 0.004g	2.65 ± 0.008h	0.13 ± 0.002g	0.23 ± 0.006h	3.84 ± 0.014i	5.46 ± 0.020h	0.42 ± 0.002b
	Saemidori	0.19 ± 0.012c	1.24 ± 0.057cd	0.02 ± 0.002efg	1.45 ± 0.050cd	0.45 ± 0.019sb	1.38 ± 0.053a	0.08 ± 0.002abcd	0.08 ± 0.004bc	1.99 ± 0.055a	3.45 ± 0.149a	0.73 ± 0.005i
	Yabukita	0.10 ± 0.000a	0.79 ± 0.011a	0.02 ± 0.002defg	0.91 ± 0.009a	0.66 ± 0.001de	2.31 ± 0.002g	0.09 ± 0.002cde	0.15 ± 0.001f	3.21 ± 0.005g	4.13 ± 0.019cd	0.28 ± 0.002a
Ariake	Yutakamidori	0.22 ± 0.006d	1.38 ± 0.034ef	0.02 ± 0.002fg	1.62 ± 0.031e	0.75 ± 0.018f	2.54 ± 0.059h	0.10 ± 0.004f	0.17 ± 0.000g	3.57 ± 0.057h	5.19 ± 0.124gh	0.46 ± 0.001c
	Saemidori	0.18 ± 0.007c	1.17 ± 0.046c	0.02 ± 0.002cdef	1.37 ± 0.039c	0.53 ± 0.019c	1.78 ± 0.065cde	0.07 ± 0.003abc	0.09 ± 0.005cd	2.48 ± 0.065cd	3.85 ± 0.147bc	0.56 ± 0.001e
	Yabukita	0.15 ± 0.002b	1.04 ± 0.020b	0.01 ± 0.004abcde	1.20 ± 0.019b	0.52 ± 0.012c	1.74 ± 0.032bcd	0.07 ± 0.001abcd	0.11 ± 0.003e	2.45 ± 0.033bcd	3.65 ± 0.073ab	0.49 ± 0.001d
Mizobe	Saemidori	0.30 ± 0.011g	1.59 ± 0.054gh	0.02 ± 0.002bcdef	1.91 ± 0.047f	0.68 ± 0.023e	2.03 ± 0.059f	0.09 ± 0.000e	0.10 ± 0.000d	2.90 ± 0.058f	4.81 ± 0.149f	0.66 ± 0.003g
	Asanoka	0.32 ± 0.003g	1.71 ± 0.017hi	0.01 ± 0.001abcd	2.04 ± 0.014f	0.66 ± 0.008de	1.91 ± 0.019ef	0.16 ± 0.008h	0.17 ± 0.003g	2.90 ± 0.011f	4.94 ± 0.035fg	0.7 ± 0.002h
	Yabukita	0.23 ± 0.004de	1.47 ± 0.010fg	0a	1.70 ± 0.010e	0.50 ± 0.000bc	1.60 ± 0.005b	0.07 ± 0.000ab	0.07 ± 0.000a	2.23 ± 0.003b	3.94 ± 0.010bc	0.76 ± 0.006j
Means ± SD		0.22±0.061	1.35 ± 0.274	0.02 ± 0.011	1.57 ± 0.316	0.61 ± 0.121	1.94 ± 0.383	0.09 ± 0.027	0.12 ± 0.047	2.83 ± 0.557	4.36 ± 0.642	0.60 ± 0.152

Table S3. Catechin content of 12 green tea breeds infused by Hot H_2O (90 °C, 1.5 min). The numerical values with different letters significantly differ ($p < 0.05$) in the same column. In "Means ± SD" row, the numerical values with different letters significantly differ ($p < 0.05$).

Area	Breed	Gallate catechins				Free catechins					Total catechins	GC/FC
		ECg	EGCg	GCg	Total	EC	EGC	(+)C	GC	Total		
Nishinoomote	Kuritawase	0.16 ± 0.036a	0.66 ± 0.142a	0.02 ± 0.008abc	0.84 ± 0.149a	0.29 ± 0.029ab	0.68 ± 0.063a	0.05 ± 0.006ab	0.06 ± 0.011ab	1.09 ± 0.079a	1.93 ± 0.279a	0.76 ± 0.086abc
	Saemidori	0.22 ± 0.040abc	1.29 ± 0.237bc	0.04 ± 0.009de	1.55 ± 0.234bc	0.30 ± 0.050ab	1.09 ± 0.176cd	0.04 ± 0.015a	0.08 ± 0.014bcd	1.52 ± 0.206bc	3.06 ± 0.538bc	1.02 ± 0.016e
	Yabukita	0.18 ± 0.015ab	1.04 ± 0.086ab	0.02 ± 0.004a	1.24 ± 0.086ab	0.28 ± 0.009ab	0.92 ± 0.032abc	0.05 ± 0.002ab	0.05 ± 0.002a	1.30 ± 0.034ab	2.54 ± 0.141ab	0.95 ± 0.049e
Chiran	Yutakamidori	0.20 ± 0.022ab	1.17 ± 0.129b	0.05 ± 0.007ef	1.42 ± 0.129b	0.45 ± 0.032c	1.55 ± 0.103e	0.08 ± 0.007cd	0.14 ± 0.012f	2.22 ± 0.124b	3.64 ± 0.306c	0.64 ± 0.027a
	Saemidori	0.20 ± 0.026ab	1.15 ± 0.142b	0.03 ± 0.005bcde	1.38 ± 0.141b	0.27 ± 0.024a	0.81 ± 0.071ab	0.05 ± 0.014ab	0.06 ± 0.006ab	1.18 ± 0.090b	2.57 ± 0.279ab	1.17 ± 0.044f
	Yabukita	0.28 ± 0.009c	1.63 ± 0.055c	0.06 ± 0.002f	1.97 ± 0.053c	0.33 ± 0.010ab	1.05 ± 0.036bcd	0.06 ± 0.005bcd	0.10 ± 0.004de	1.54 ± 0.043c	3.51 ± 0.117c	1.28 ± 0.006f
Ariake	Yutakamidori	0.20 ± 0.036ab	1.16 ± 0.213b	0.04 ± 0.005bcde	1.40 ± 0.207b	0.45 ± 0.045c	1.52 ± 0.202e	0.07 ± 0.006cd	0.11 ± 0.013e	2.15 ± 0.217b	3.55 ± 0.517b	0.65 ± 0.033ab
	Saemidori	0.21 ± 0.014ab	1.17 ± 0.070b	0.03 ± 0.004abcd	1.41 ± 0.071b	0.31 ± 0.015ab	1.12 ± 0.063cd	0.05 ± 0.006ab	0.07 ± 0.006abc	1.55 ± 0.067b	2.96 ± 0.168b	0.9 ± 0.014de
	Yabukita	0.21 ± 0.014abc	1.30 ± 0.087bc	0.04 ± 0.005cde	1.54 ± 0.087bc	0.33 ± 0.008ab	1.18 ± 0.045cd	0.06 ± 0.002abc	0.09 ± 0.005cde	1.66 ± 0.045bc	3.21 ± 0.159bc	0.93 ± 0.029e
Mizobe	Saemidori	0.19 ± 0.014ab	0.93 ± 0.085ab	0.02 ± 0.001a	1.13 ± 0.081ab	0.32 ± 0.013ab	1.02 ± 0.085bcd	0.05 ± 0.003ab	0.06 ± 0.002a	1.44 ± 0.078ab	2.57 ± 0.194ab	0.79 ± 0.016bcd
	Asanoka	0.24 ± 0.020bc	1.17 ± 0.106b	0.02 ± 0.004abc	1.44 ± 0.105b	0.31 ± 0.010ab	0.99 ± 0.036bcd	0.08 ± 0.006b	0.08 ± 0.003bcd	1.47 ± 0.043b	2.90 ± 0.181bc	0.98 ± 0.044e
	Yabukita	0.22 ± 0.011abc	1.29 ± 0.059bc	0.02 ± 0.002ab	1.53 ± 0.059bc	0.36 ± 0.011b	1.22 ± 0.047d	0.06 ± 0.005abc	0.06 ± 0.004ab	1.70 ± 0.053bc	3.22 ± 0.123bc	0.9 ± 0.029cde
Means ± SD		0.21 ± 0.031	1.16 ± 0.231	0.03 ± 0.012	1.36 ± 0.293	0.33±0.059	1.10 ± 0.255	0.06 ± 0.013	0.08 ± 0.027	1.57 ± 0.313	2.97 ± 0.506	0.90 ± 0.182
